# Subacute Toxicity Study of Nicotinamide Mononucleotide via Oral Administration

**DOI:** 10.3389/fphar.2020.604404

**Published:** 2020-12-15

**Authors:** Yingnan You, Yang Gao, Han Wang, Jingshu Li, Xiang Zhang, Zhengjiang Zhu, Nan Liu

**Affiliations:** ^1^ Interdisciplinary Research Center on Biology and Chemistry, Shanghai Institute of Organic Chemistry, Chinese Academy of Sciences, Shanghai, China; ^2^ University of Chinese Academy of Sciences, Beijing, China; ^3^ GeneChic Genomics, Shanghai, China

**Keywords:** nicotinamide mononucleotide, subacute toxicity, mice, beagle dogs, oral administration

## Abstract

Nicotinamide mononucleotide (NMN), a key precursory metabolite of NAD^+^, has been shown to elevate the cellular level of NAD^+^ and ameliorate various age-related diseases. Despite these progresses, systemic evaluation pertaining to the subacute toxicity of NMN remains to be determined. Here, we examine the subacute toxicity of NMN in mice and beagle dogs. Mice were gavaged with a saturated concentration of NMN solution at the maximum intragastric dose once or twice per day for 7 days. Dogs were gavaged twice per day for 14 days. In mice, NMN administrated once per day for 7 days is well tolerated with minimal deleterious effects. Upon higher dosage, we observe slightly increased level of alamine aminotransferase, while other biomarkers remain unchanged. Consistently, administration of NMN in beagle dogs only results in mild increases in creatinine and uric acid. Together, our study highlights the safety of NMN, providing a possible safe dose range for oral administration of NMN.

## Introduction

Aging is characterized by the progressive decline in cellular and organismal functions that lead to reduction of fitness and increased risks to diseases and death (Lopez-Otin et al., 2013). Decline in metabolic homeostasis represent a prominent hallmark of aging (Lopez-Otin et al., 2016). NAD^+^ is the dominant metabolite for cells, which is essentially involved in a wide-range of biological processes (Braidy et al., 2019). In rodents and humans, studies have revealed that the NAD^+^ level declines with age in critical organs, such as heart (Braidy et al., 2011), pancreas (Yoshino et al., 2011), adipose tissue (Yoshino et al., 2011), skeletal muscle (Yoshino et al., 2011), liver (Yoshino et al., 2011), skin (Massudi et al., 2012), and brain (Zhu et al., 2015). Importantly, it has been demonstrated that increasing cellular of NAD^+^ extends lifespan in yeast (Anderson et al., 2002), worms (Mouchiroud et al., 2013), flies (Balan et al., 2008) and mice (Zhang et al., 2016). Thus, enhancing NAD^+^ biosynthesis by pharmaceutical approach is expected to provide preventive effects on various pathophysiological changes associated with disease as well as during the natural process of aging.

Nicotinamide mononucleotide (NMN), a key precursor metabolite for NAD^+^ biogenesis, could be such a candidate (Partridge et al., 2020). Notably, administration of NMN has been reported to have remarkable therapeutic effects on age-related diseases. For examples, NMN maintains the neural stem/progenitor cell population (Stein and Imai, 2014), restores skeletal muscle mitochondrial function (Gomes et al., 2013), arterial function (de Picciotto et al., 2016), and ameliorates cognitive function (Kiss et al., 2019) in aged and diseased mouse models.

Mounting evidence associates the exogenously administered NMN with improved adult fitness, For instance, it is reported that long-term administration of NMN could mitigate age-associated physiological decline in mice (Mills et al., 2016) and short-term administration of NMN could induce similar reversal of the glucose intolerance induced by obesity (Uddin et al., 2016). But the proper dosage of NMN is only beginning to be investigated. Mouse models have routinely used the NMN dosage of 500 mg/kg/d, which have thus far been safe (Yoshino et al., 2018). Moreover, a preliminarily report has shown that the single oral administration of 100, 250, and 500 mg NMN per day appear to be safe in healthy human subjects (Irie et al., 2020). Despite these progresses, systemic evaluation regarding the subacute toxicity of NMN remain to be determined. In the present research, we examine the subacute toxicity of NMN in mice and beagle dogs. Mice were gavaged with saturated concentration of NMN solution at the maximum intragastric dose once or twice per day for 7 days. Dogs were gavaged twice per day with 10 ml saturated concentration of NMN solution for 14 days. We subsequently assess the hepatotoxicity and nephrotoxicity of animals following NMN treatment. Despite high-intake of NMN employed throughout this study, our data indicates that oral administration of NMN has minor adverse effects on animals.

## Materials and Methods

### Ethics Statement

All animal procedures have been reviewed and approved by the Institutional Animal Care and Use Committee at Chinese Academy of Sciences and are in accordance with the Guide for the Care and Use of Laboratory Animals of Chinese Academy of Sciences. All efforts were made to minimize the suffering of the animals.

### Animal Experimentation

The subacute toxicity was tested in healthy male C57BL6J mice aged 8 weeks with weight between 20 and 30 g. NMN was supplied from Shokou Life Tech (Shokou, Japan) and dissolved in sterile water. Mice were randomly divided into four groups: mice gavaged with water once daily; mice gavaged with NMN once daily; mice gavaged with water twice daily; and mice gavaged with NMN twice daily. three to five mice were in each group. Blood was collected via cardiac puncture. After collection of the whole blood, allow the blood to clot by leaving it undisturbed at room temperature. Remove the clot by centrifuging at 3000 g for 15 min at 4 °C. The resulting supernatant is designated serum. Liver and kidney were collected after NMN administration.

10 beagle dogs aged 4 years with weight between 9 and 11 kg were randomly divided into two groups: dogs were gavaged twice per day each with 10 ml water and dogs were gavaged twice per day each with 10 ml NMN; serum was collected before and after NMN administration.

### General Observation

Cage-side examinations for apparent signs (behavior, mental status, gland secretion, respiration status, feces characters, genitals, and death) of toxicity or injury were conducted once a day after daily drug exposure.

### Histopathological Evaluations

Kidney and liver were harvested and immersed in fixative (4% paraformaldehyde). Tissues were dehydrated by ethanol solution in serial concentrations, cleared with xylene and embedded with paraffin. Paraffin blocks were sectioned with microtome into 5 μm thickness and each tissue section was stained with hematoxylin and eosin followed by microscopic examination by light microscope (Nikon Ds-Ri2).

### PolyA-Selected mRNA-Seq

RNA isolation was followed in accordance with manufacturer’s instruction (Takara Bio, Japan). RNA was re-suspended in DEPC-treated RNase free water (Invitrogen, United States). TURBO DNA free kit was used to remove residual DNA contamination according to manufacturer’s instruction (Invitrogen, United States). A concentration of 1 μg of total RNA was used to generate sequencing library using Vazyme VAHTS mRNA-seq v3 library Prep Kit for Illumina. The library quality was checked by Bioanalyzer 2,100 (Agilent, United States). The quantification was performed by qRT-PCR with a reference to a standard library. The libraries were pooled together in equimolar amounts to a final 2 nM concentration. The normalized libraries were denatured with 0.1 M NaOH (Sigma, United States). Pooled denature libraries were sequenced on the Illumina NextSeq 550 or Illumina Hiseq Xten. Sequencing reads were mapped to the reference genome mm19 with STAR by default parameter. The read counts for each gene were calculated by HTSEQ_COUNT (version 0.11.0.) with parameters “-m intersection-strict -s no.” The count files were used as input to R package DESeq2 (version 1.24.0.) for normalization.

### Serum Biochemistry Evaluation

Serum was analyzed for the function of liver, function of kidney, insulin and blood lipids using automatic hematology analyzer (Beckman AU5811). Biochemistry and hematological analyses were performed in ADICON clinical laboratories (Shanghai, China).

### NAM Measurements of Serum

400 μL MeOH and 400 μL ACN were added to 100 μL serum. Incubated for 1 h at −20 °C, centrifuged for 15 min at 13,000 rpm and 4 °C. Took the supernatant and evaporated to dryness at 4 °C. Reconstituted with 100 μL ACN/H2O (volume/volume, 1:1), vortexed for 30 s, sonicated for 10 min (4 °C water bath). Centrifuged for 15 min at 13,000 rpm and 4 °C. Supernatants were analyzed by LC-MS. The LC–MS analysis was performed using an UHPLC system (1,290 series, Agilent Technologies) coupled to a triple quadrupole mass spectrometer (Agilent 6,495 QqQ, Agilent Technologies). Chromatographic separation was performed on the Merck ZIC-pHILIC column (100 × 2.1 mm, 5 μm). The column was maintained at room temperature, and the flow rate was 0.2 ml/min. Mobile phase A was 100% H2O, 25 mM CH3COONH4, and 25 mM NH4OH, and mobile phase B was 100% acetonitrile. The column was eluted with a linear gradient system (B %): 0 min, 80%; 1 min, 80%; 3 min, 65%; 7 min, 50%; 7.1, 20%; 9.5 min, 20%; 9.6 min, 80%; 13 min, 80%. Optimized MRM transition 123.1/53.1 in positive mode was utilized for quantifying NAM.

### NAD^+^ Measurements of Liver

Assays were assembled according to the instruction of manufacturers (Beyotime, China).

### Statistical Analysis

Data were calculated and analyzed with Graph Pad Prism version 8.1.1 (Prizm, United States). All values were presented as mean ± standard error and calculated using an unpaired Student t-test.

## Results

### Oral Administration of NMN Substantially Increases Tissue NAD^+^ Levels With Minimal Adverse Effects

**FIGURE 1:**
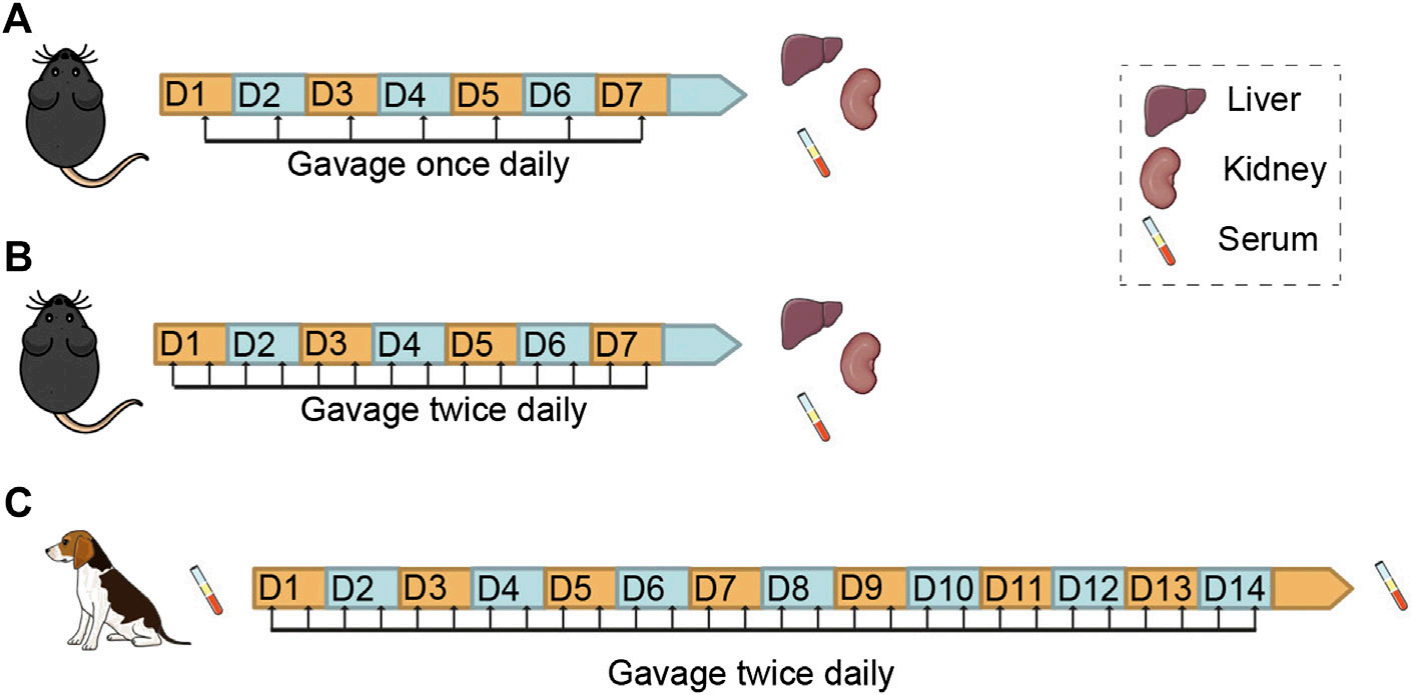
Study Design for the Examination of Subacute Toxicity of Nicotinamide Mononucleotide (NMN) in Mice and Dogs Schemes showing the route, frequency and duration of NMN administration and sampling in mice. **(A,B)** and beagle dogs **(C)**.

**FIGURE 2:**
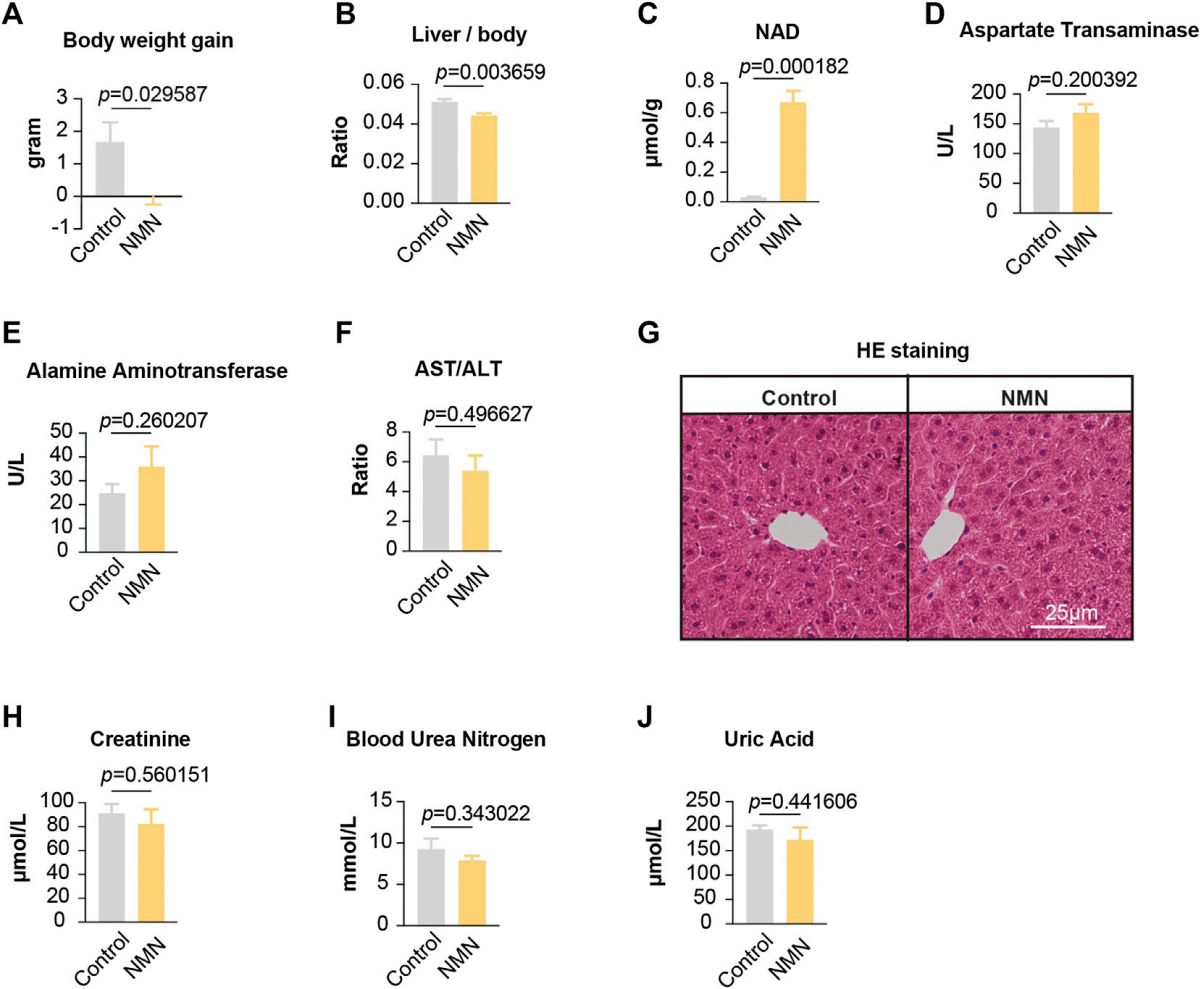
Data for mice treated with NMN once per day **(A)**The body weight was decreased after NMN administration. **(B)** The ratio between liver weight and body weight was decreased after NMN administration **(C)** Liver NAD^+^ levels were increased after NMN administration. **(D–F)** Liver function was analyzed after NMN administration. **(G)** Photomicrograph of liver sections from NMN-administered mice exhibited normal hepatocytes (H&E stain). **(H–J)** Kidney function were analyzed after NMN administration.

To evaluate the subacute toxicity of NMN, we treated 8-week-old male mice with a saturated concentration of NMN solution (67 mg/ml) at the maximum intragastric dose (20 ml/kg). After 24 h of NMN supplementation, all animals remained alive and exhibited no difference in fur and eye color. Compared to control animals, NMN-treated mice had similar locomotion activity with no occurrence of piloerection and diarrhea. Following the same NMN feeding scheme, we gavaged the mice once per day (1,340 mg/kg/d) for a week (Figure 1A). After 7 days of NMN administration, animals were viable with no obvious behavioral and morphological deficits. The body weight from NMN-treated mice was decreased compared to control animals (Figure 2A). Similarly, the ratio between liver and body weight was also decreased upon NMN treatment (Figure 2B).

To confirm whether our feeding scheme had an impact on the level of NAD^+^ in tissues, we measured liver NAD^+^. Our data showed that NAD^+^ in liver was dramatically increased (Figure 2C), suggesting that oral administration of NMN efficiently boosts NAD^+^ in tissue. To determine the effects of NMN on liver, we assessed liver function and anatomy (Figures 2D–G). Measurements of Aspartate Transaminase (AST), Alamine Aminotransferase (ALT), and AST/ALT found no significant difference (Figures 2D–F). Photomicrograph of liver sections from NMN-administered mice exhibited normal hepatocytes (Figure 2G). Furthermore, we examined the kidney function by assessing creatinine, blood urea nitrogen, and uric acid. These analyses showed comparable levels between control and NMN-treated animals (Figures 2H–J). Taken together, our data demonstrate that oral gavage of NMN substantially elevates NAD^+^ in tissue with no significant side effects on liver and kidney.

### Gavage of Higher NMN Mildly Elevates Alamine Aminotransferase Levels

**FIGURE 3:**
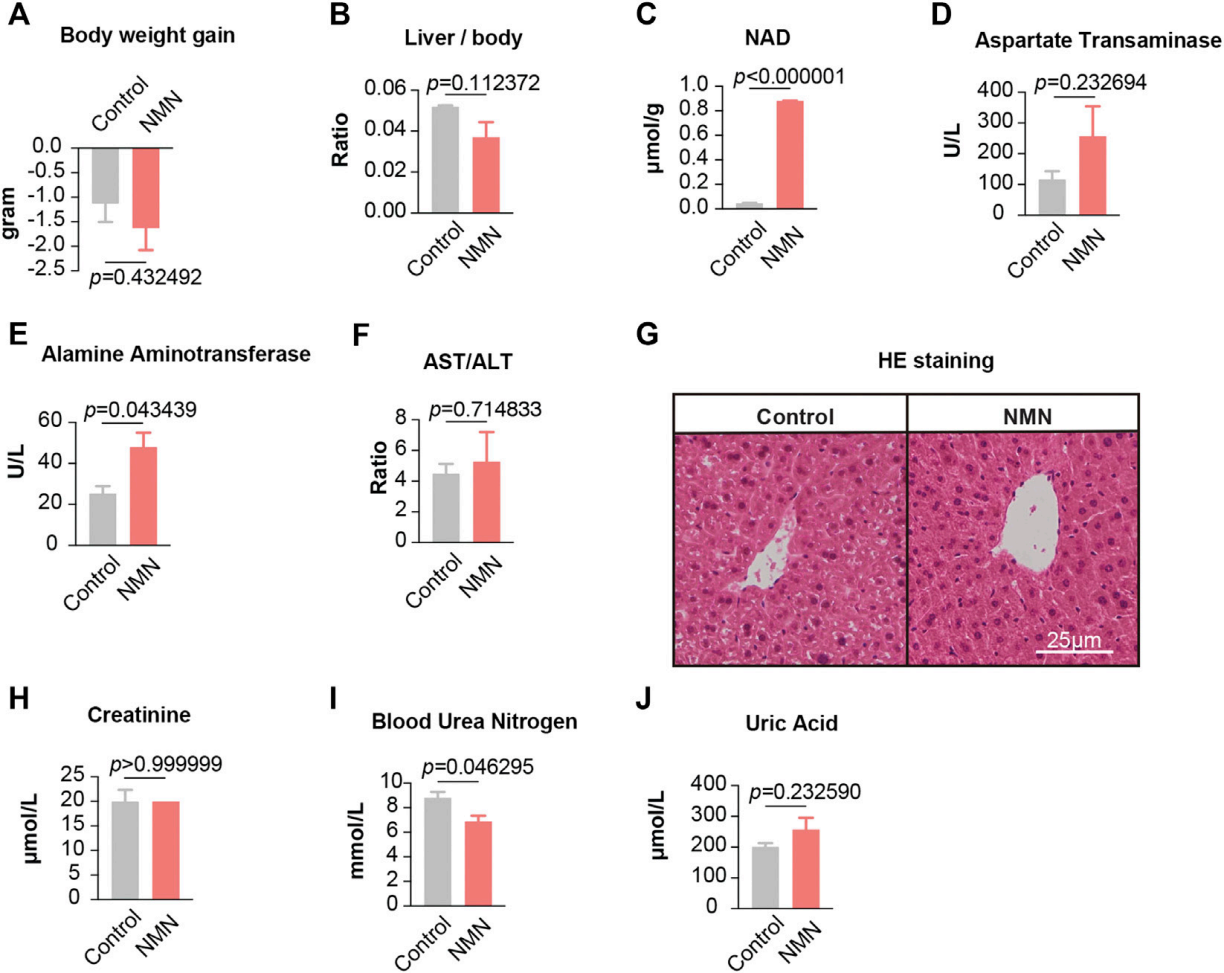
Data for mice treated with NMN twice per day **(A)** The body weights were measured before and after higher NMN administration. **(B)** The ratio between liver weight and body weight was measured after higher NMN administration. **(C)** Liver NAD^+^ levels were increased after higher NMN administration. **(D–F)** Liver function were analyzed after higher NMN administration. **(E)** Alamine aminotransferase was evaluated in higher NMN-administered mice. **(G)** Photomicrograph of liver sections from higher NMN-administered mice exhibited normal hepatocytes (H&E stain). **(H–J)** Kidney function were analyzed after higher NMN administration. **(I)** Blood urea nitrogen was decreased in 1340 mg/kg/d NMN-administered mice.

Since gavage once per day demonstrates minimal adverse effects, we further increased the frequency of gavage to two times per day (2,680 mg/kg/d) (Figure 1B). After 7 days, all animals survived but had a comparable weight loss in both control and NMN-treated mice (Figure 3A). Though the extent of decrease did not reach statistical significance, the ratio between liver and body weight was decreased in NMN-treated mice (Figure 3B). Consistently, NAD^+^ levels in liver were substantially increased (Figure 3C). Unlike gavage once per day, alamine aminotransferase levels were elevated upon the use of higher dosage of NMN (Figure 3E). Histopathological examinations, however, found no defect in liver anatomy (Figure 3G). Similarly, metabolites that reflect kidney function were examined (Figures 3H–J). Although blood urea nitrogen concentration was slightly decreased in the NMN-treated mice (Figure 3I), there was no difference in the serum concentration of creatinine and uric acid between control and NMN-treated animals, suggesting minimal adverse effects on kidney function. Taken together, despite the fact that alamine aminotransferase is mildly increased, overall physiological functions of liver and kidney remain intact when higher dosage of NMN is employed.

### Transcriptomic Analysis in NMN Administrated Mice Liver

**FIGURE 4:**
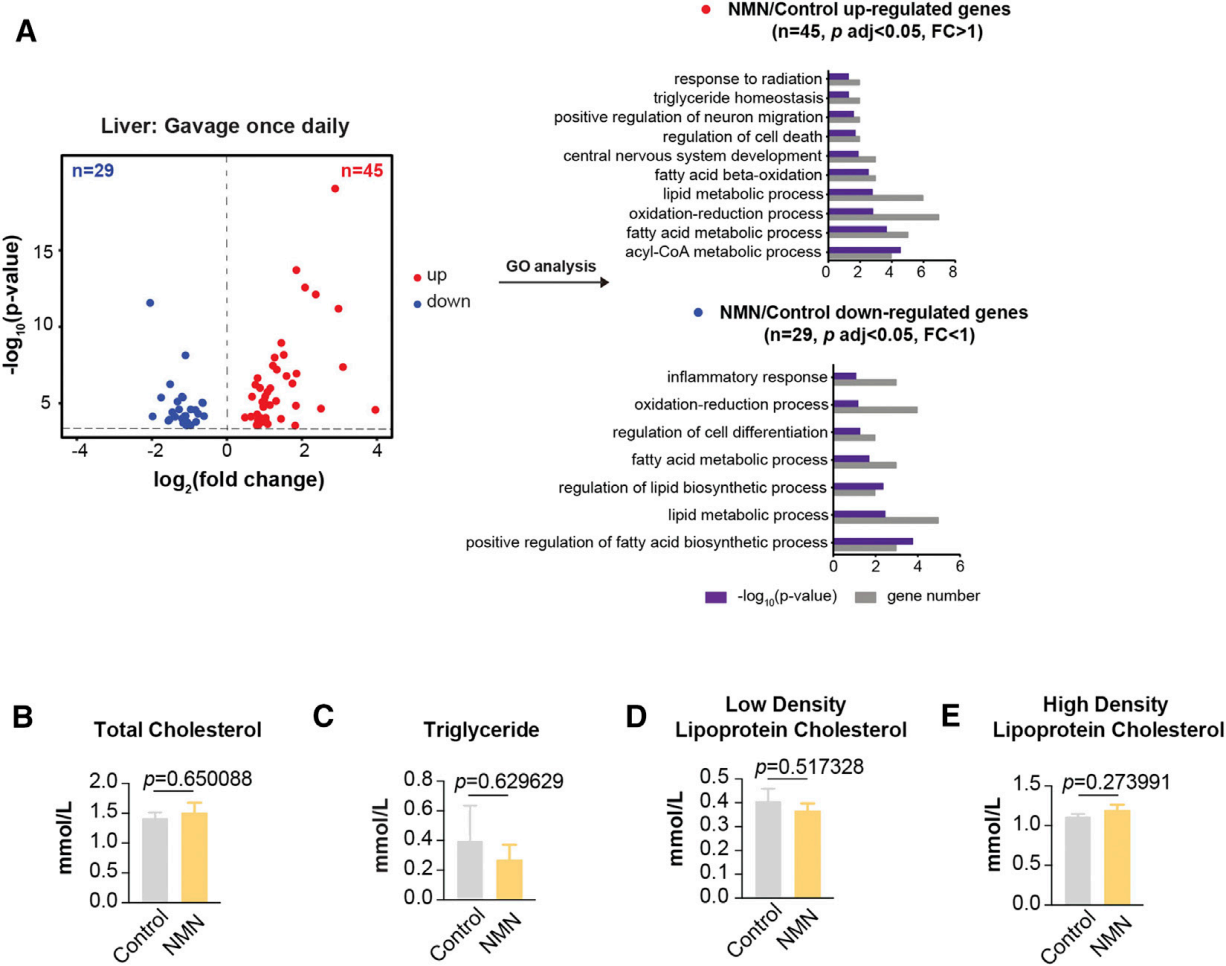
Transcriptomic Analysis in 1340 mg/kg/d NMN-Administrated Mice Liver **(A)** Volcano plot shows that genes are differentially expressed in 1340 mg/kg/d NMN-administrated mice compared to control in liver **(left panel)**. Differential expression analysis was performed by DESeq2 from four biological replicates. 45 genes were found to be upregulated (*p* < 0.05, FC > 1), 29 genes were down regulated (*p* < 0.05, FC < 1). Gene ontology (GO) analysis were performed by David **(right panel)**. The result shows lipid metabolic process being the biological processes significantly enriched for genes up and down regulated in 1340 mg/kg/d NMN administration group. The bar graphs represent–log10 (*p* value) and gene counts in each pathway. **(B–E)** Blood lipids were measured after 1340 mg/kg/d NMN administration.

Using liver tissue, we performed transcriptomic analysis by polyA-selected RNA sequencing (RNA-seq). In mice gavaged once per day, only a subset of 74 genes were significantly altered in liver (*p* < 0.05), with 45 genes up-regulated and 29 genes down-regulated (Figure 4A). Gene ontology (GO) analysis showed lipid metabolic process being the biological processes significantly enriched (Figure 4A). Given this result, we measured profile of select lipids. As noted, blood lipids were comparable between control and NMN-treated animals (Figures 4B–E).

**FIGURE 5:**
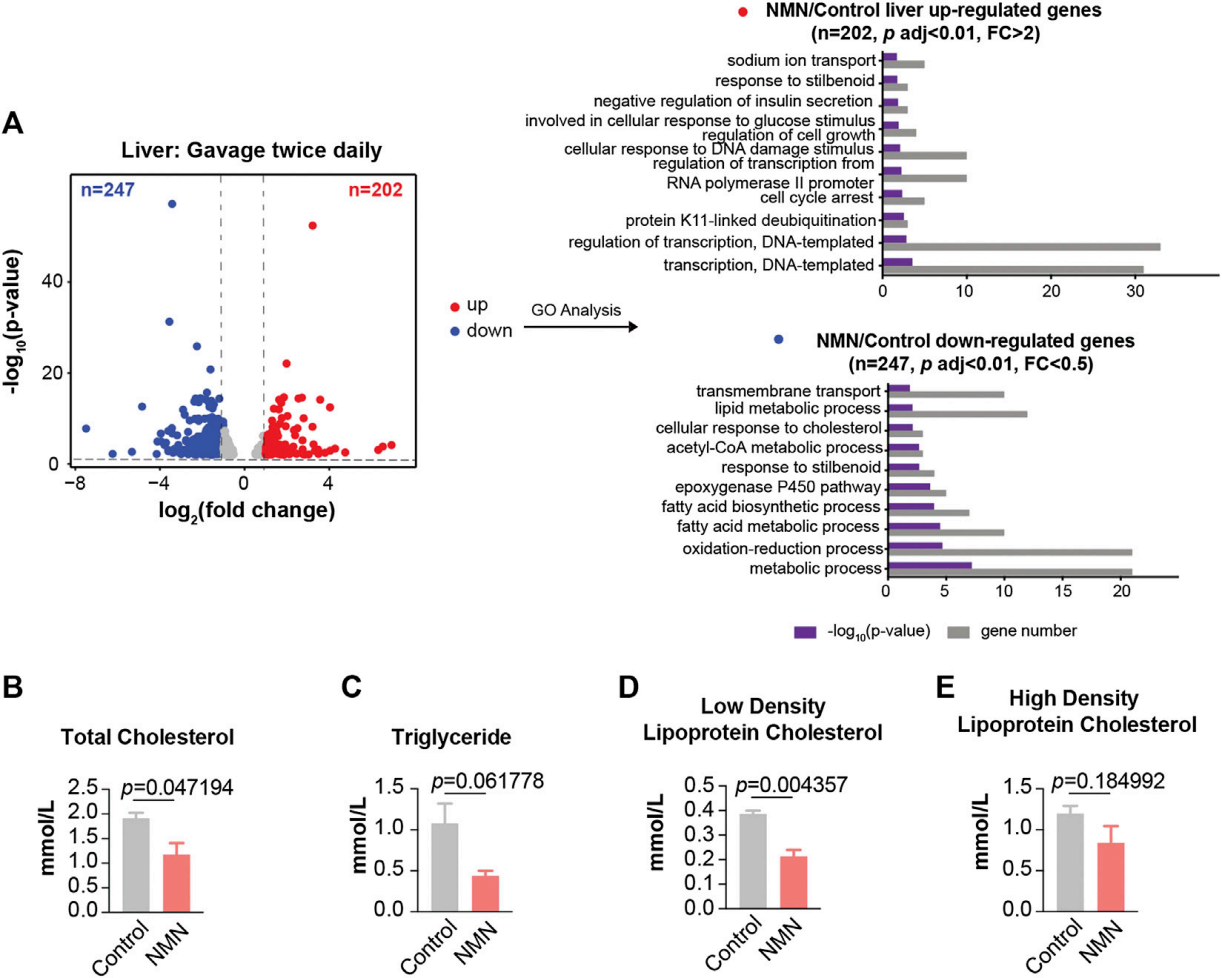
Transcriptomic Analysis in 2680 mg/kg/d NMN-Administrated Mice Liver **(A)** Volcano plot shows that genes are differentially expressed in 2680 mg/kg/d NMN-administrated mice compared to control in liver **(left panel)**. Differential expression analysis was performed by DESeq2 from three biological replicates. 202 genes were found to be upregulated (*p* < 0.01, FC > 2), 247 genes were down regulated (*p* < 0.01, FC < 0.5). Gene ontology (GO) analysis were performed by David **(right panel)**. The result shows lipid metabolic process being the biological processes significantly enriched for genes down regulated in 2680 mg/kg/d NMN administration group. The bar graphs represent–log10 (*p* value) and gene counts in each pathway. **(B–E)** Blood lipids were measured after 2680 mg/kg/d NMN administration. **(B)** Total Cholesterol was decreased in 2680 mg/kg/d NMN-administered mice. **(D)** Low density lipoprotein cholesterol was decreased in 2680 mg/kg/d NMN-administered mice.

In mice that received higher NMN dosage, a broad change in the transcriptome was observed, in which 449 genes were significantly altered in liver (*p* < 0.01; fold change > 2 or fold change < 0.5) (Figure 5A). GO analysis also indicated lipid metabolic process being prominently highlighted. Measurements of blood lipids, including total cholesterol, triglyceride, and low density lipoprotein cholesterol, were significantly decreased in NMN-treated animals (Figures 5B–E). This data suggests that the effect of NMN on blood lipid is dose-dependent.

### Transcriptomic Analysis in NMN Administrated Mice Kidney

**FIGURE 6:**
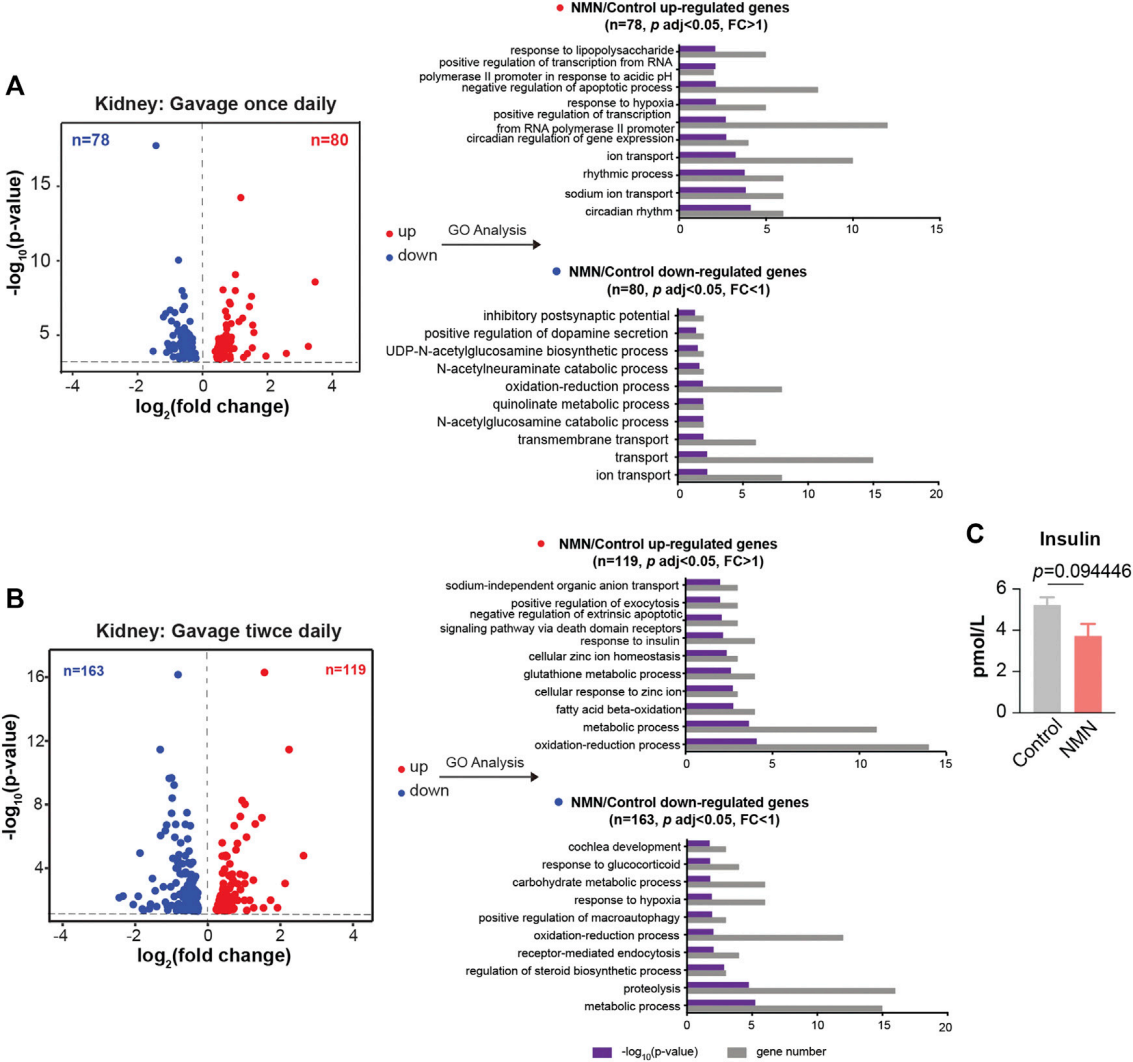
Transcriptomic Analysis in NMN Administrated Mice Kidney. **(A)** Volcano plot shows that genes are differentially expressed in 1340 mg/kg/d NMN-administrated mice compared to control in kidney **(left panel)**. Differential expression analysis was performed by DESeq2 from four biological replicates. 80 genes were found to be upregulated (*p* < 0.05, FC > 1), 79 genes were down regulated (*p* < 0.05, FC > 1). Gene ontology (GO) analysis were performed by David **(right panel)**. **(B)**. Volcano plot shows that genes are differentially expressed in 2680 mg/kg/d NMN-administrated mice compared to control in kidney **(left panel)**. Differential expression analysis was performed by DESeq2 from three biological replicates. 119 genes were found to be upregulated (*p* < 0.05, FC > 1), 163 genes were down regulated (*p* < 0.05, FC < 1). Gene ontology (GO) analysis were performed by David **(right panel)**. Gene ontology (GO) analysis (performed by David) shows response to insulin being the biological processes significantly enriched for genes down regulated in 2680 mg/kg/d NMN administration group. **(C)** The insulin level in serum was measured after NMN administration.

We further extended RNA-seq analysis to kidney. In mice gavaged once daily, GO analysis showed that up-regulated genes were enriched in circadian rhythm and sodium ion transport, while down-regulated genes were enriched in ion transport (Figure 6A). In mice gavaged twice daily, up-regulated genes were enriched in oxidation-reduction process (Figure 6B). Interestingly, “response to insulin” was enriched for genes up-regulated (Figure 6B). Since it has been reported that NMN-administered mice show improved insulin sensitivity (Yoshino et al., 2011), we conducted insulin test in serum, which revealed a decreased concentration of insulin (Figure 6C). Down-regulated genes, on the other hand, were enriched in the metabolic process (Figure 6B).

### The Effect of Oral 1,340 mg/d NMN-Administration on Liver Function, Kidney Function and Blood Lipids in Beagle Dogs

**FIGURE 7:**
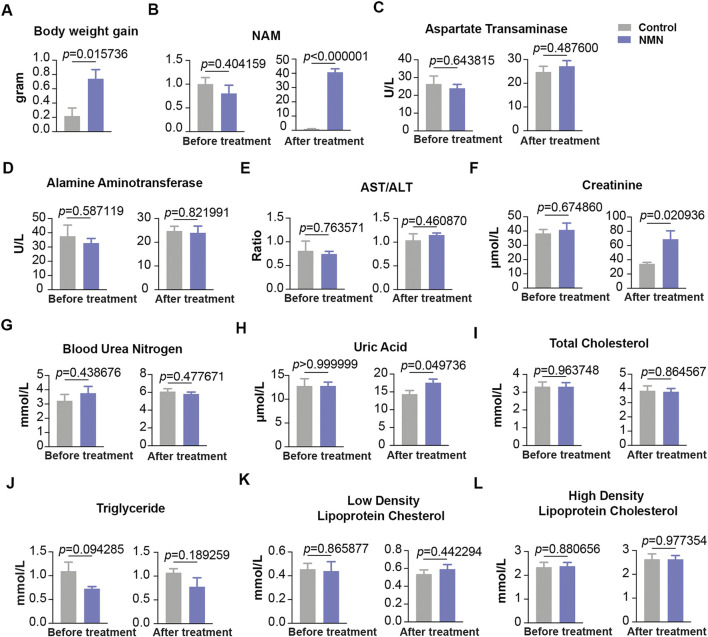
The effect of oral 1340 mg/d NMN-administration on liver function, kidney function and blood lipids in beagle dogs **(A)** Body weights were measured before and after NMN administration. **(B)** Serum NAM levels were increased after NMN administration. **(C–E)** Liver function was analyzed after NMN administration. **(F–H)** Kidney function was analyzed after NMN administration. **(F)** Creatinine was evaluated in 1340 mg/kg/d NMN-administered dogs. **(H)** Uric acid was evaluated in 1340 mg/kg/d NMN-administered dogs. **(I–L)** Blood lipids were measured before and after NMN administration.

To gain insight of the toxicity of NMN in large mammal, we design oral gavage test using beagle dogs. 10 beagle dogs were randomly divided into two groups (Table 1). Dogs were gavaged twice per day with 10 ml saturated concentration of NMN solution for 14 days (1340 mg/d) (Figure 1C). During the experiment, animals were found to be in good condition, with normal autonomic activities and clean coats. After two weeks of NMN administration, the body weight of NMN-treated dogs was increased than that of the control dogs (Figure 7A). Since NAD^+^ has exceedingly low level in plasma and urine (Trammell et al., 2016), we measured NAM in serum which has been shown be converted from exogenous NMN (Liu et al., 2018). Notably, NAM levels were dramatically increased in NMN-treated dogs (Figure 7B). To evaluate the hepatotoxicity and nephrotoxicity, blood metabolites were analyzed (Figures 7C–H). In NMN-treated, creatinine and uric acid were increased, indicating kidney response. Blood lipids were measured (Figures 7I–L), which showed comparable levels between control and NMN-treated dogs.

**TABLE 1:** The age, weight and groups of beagle dogs.

No	Group	Age (y)	Weight (kg)
1	Control	4	10.0
2	Control	4	10.0
3	Control	4	9.0
4	Control	4	10.3
5	Control	4	9.9
6	NMN-treated	4	10.7
7	NMN-treated	4	10.1
8	NMN-treated	4	11.0
9	NMN-treated	4	9.0
10	NMN-treated	4	10.0

## Discussion

Emerging studies have heightened the fact that the cellular level of NAD^+^ decreases with age, predisposing individuals to physiological decline as well as late-onset diseases (Rajman et al., 2018). Thus, enhancing NAD^+^ availability by supplementing precursory metabolites, such as NMN and NR, promises to ameliorate a broad spectrum of age-associated deficits (Partridge et al., 2020). However, prior to the application in humans, systemic evaluation of the NMN toxicity especially in model organisms and large mammals remains to be determined. In the present study, we assess the subacute toxicity of NMN in mice and dogs.

Previous studies in mice apply 100–500 mg/kg/d of NMN, equivalent surface area dose for a human adult being approximately 0.5–2.5 g, depending on the weight of individuals. To examine subacute toxicity, we substantially increase NMN dosage to 1,340 mg/kg/d and 2680 mg/kg/d in mouse models. To our knowledge, such high intake of NMN for animal test has not yet been used. As noted, administrating 1,340 mg/kg/d NMN for 7 days is well tolerated with no deleterious effects. However, as the dose doubled, we observe slightly increased level of alamine aminotransferase among all the biomarkers examined. On the other hand, administrating 1340 mg/d NMN in beagle dogs only results in mild increases in creatinine and uric acid, while other biomarkers remain unchanged. Taken together, our data indicates that high-dose and short-term oral administration of NMN has mild or minimal deleterious effects.

Interestingly, our analysis reveals that NMN administration has beneficial effects to lower lipid and insulin levels. Although it has been shown that long-term supplementation of NMN improves glucose intolerance and lipid profiles in a mouse model of age-induced type-2 diabetes (Yoshino et al., 2011), our data suggests that high-dose NMN within a week is sufficient to reduce blood lipids and improve insulin sensitivity. NMN therefore has the potential to be implemented as safe therapeutic agent against age-associated physiological decline and disease. Our findings from this short-term administration study provide a possible safe dose range for oral administration of NMN, hoping to translate the results to humans.

## Data Availability Statement

The datasets presented in this study can be found in online repositories. The names of the repository/repositories and accession number(s) can be found below: https://www.ncbi.nlm.nih.gov/geo/GSE157594.

## Ethics Statement

The animal study was reviewed and approved by Institutional Animal Care and Use Committee at Chinese Academy of Sciences.

## Author Contributions

YY and NL contributed to concept and design of the study. YG and ZZ contributed to the detection of NAM. YY, JL, and HW collected the mouse samples. NL, YY, and XZ collected the dog samples. YY performed the statistical analysis. NL and YY wrote the manuscript.

## Funding

This work was supported in part by grants from the National Key Research and Development Program of China (2016YFA0501900) and the China National Natural Science Foundation (91849109) (to NL).

## Conflict of Interest

The authors declare that the research was conducted in the absence of any commercial or financial relationships that could be construed as a potential conflict of interest.
